# Chemical Composition and Hypotensive Effect of *Campomanesia xanthocarpa*

**DOI:** 10.1155/2017/1591762

**Published:** 2017-05-11

**Authors:** Liane Santariano Sant'Anna, Liara Merlugo, Catrine Santos Ehle, Jessica Limberger, Maquelen Blanco Fernandes, Marí Castro Santos, Andreas Sebastian Loureiro Mendez, Fávero Reisdorfer Paula, Cleci Menezes Moreira

**Affiliations:** ^1^Universidade Federal do Pampa, Uruguaiana, RS, Brazil; ^2^Faculdade de Farmácia, Universidade Federal do Rio Grande do Sul, Porto Alegre, RS, Brazil

## Abstract

*Campomanesia xanthocarpa* is known in Brazil as Guabiroba and is popularly used for various diseases, such as inflammatory, renal, and digestive diseases and dyslipidemia. The aim of the study was to analyze the chemical composition and investigate the effects of aqueous extract of* C. xanthocarpa* on the blood pressure of normotensive rats, analyzing the possible action mechanism using experimental and in silico procedures. The extract was evaluated for total phenolic compounds and total flavonoid content. The chemical components were determined by HPLC analyses. Systolic and diastolic blood pressure and heart rate were measured with extract and drugs administration. The leaves of* C. xanthocarpa* presented the relevant content of phenolics and flavonoids, and we suggested the presence of chlorogenic acid, gallic acid, quercetin, and theobromine. The acute administration of aqueous extract of* C. xanthocarpa* has a dose-dependent hypotensive effect in normotensive rats, suggesting that the action mechanism may be mediated through the renin-angiotensin system by AT1 receptor blockade and sympathetic autonomic response. Docking studies showed models that indicated an interaction between chlorogenic acid and quercetin with the AT1 receptor (AT1R) active site. The findings of these docking studies suggest the potential of* C. xanthocarpa* constituents for use as preventive agents for blood pressure.

## 1. Introduction

Essential hypertension is a highly prevalent pathological condition that is considered one of the most relevant cardiovascular risk factors and is an important cause of morbidity and mortality around the world. Major effects on renal and cardiovascular physiology attributed to angiotensin II are mediated through the AT1R [1]. Chronic activation of the AT1R can lead to disease states, including hypertension, cardiac arrhythmia, stroke, diabetic nephropathy, and metabolic disorders [2–4]. The AT1a receptor is well expressed in most cardiovascular tissues and is the principal regulator of blood pressure [5], which is effectively treated using AT1R blockers [4, 6, 7].

Many synthetic drugs have been widely used for the treatment of hypertension, but herbal medicines still remain a popular choice. The abundant use of these medicinal plants has led to extensive research in this area to determine their potential efficacy, and modern cardiovascular drugs are now available as natural products [8].

In Brazil,* Campomanesia xanthocarpa* (Myrtaceae), popularly known as “Guabiroba,” possesses a wide spectrum of physiological effects. The leaves of this plant are used as an infusion in folk medicine to treat inflammatory, urinary, and rheumatic diseases, and hypercholesterolemia [9]. Moreover, it is empirically used for weight loss and for the control of many conditions associated with obesity [10]. Scientific studies demonstrate that* C. xanthocarpa* extract presents mutagenic potential, synergistic effects that result in comutagenic activity [11], has antiproliferative and genotoxic activities using the in vivo* Allium cepa* root-tip cell test and an increase in the frequency of chromosome aberrations [12], has antiplatelet, antithrombotic, and fibrinolytic activities, may be effective in preventing thrombus formation through several pathways [13], reducing blood cholesterol levels [14], has a gastroprotective effect [15], and reduces oxidative stress and inflammatory processes [16, 17]; it may therefore have therapeutic applications.

Natural biological active compounds in plants have a significant role in vegetable defense mechanism and are also important for their unambiguous physiological actions in human body. Due to their therapeutic properties, secondary metabolites (flavonoids, alkaloids, tannins, saponins, and terpenoids) are becoming a part of the integrative health care system as supportive and alternative medicines [18]. Thus, knowing the chemical composition of these medicinal plants is very important.

Phytochemical analysis from the leaves of* C. xanthocarpa* extract indicated the presence of flavonoids, saponins, tannins, and terpenes [12, 14, 15]; these compounds have known biological potential. Thus, the objective of this study is to investigate the chemical composition of* C. xanthocarpa* extract, evaluate the hemodynamic parameters, and investigate the action mechanism of the aqueous extract in rats by experimental and in silico procedures (docking analysis).

## 2. Material and Methods

### 2.1. Plant Material

*Campomanesia xanthocarpa* was collected in July 2014 in Uruguaiana/RS (Brazil). The materials were identified, and a voucher specimen was deposited at the Herbarium of Universidade Federal do Pampa–São Gabriel/RS.

### 2.2. Extract Preparation

The leaves were selected, dried at a temperature of 40°C, ground, and submitted to an infusion using distilled water at a temperature of 100°C (1 : 10, plant : solvent), used in chromatographic assays.

For biological analysis, the infusions were oven-dried, reduced to powder, and rediluted in 0.9% saline solution for administration.

### 2.3. Characterization of Plants and Extracts

#### 2.3.1. Total Phenolic Content

The Folin-Ciocalteu colorimetric method with some modifications was used to determine the total phenolic content [19]. To a test tube were added 100 *μ*L of the sample, 500 *μ*L of Folin-Ciocalteu, and 6 mL of water; the tube was agitated and left to rest for one minute. Two milliliters of Na_2_CO_3_ solution 15% was added after alkalizing the medium, and the volume was completed to 10 mL with distilled water. It was read in a spectrophotometer Perkin Elmer UV-VIS Lambda 35® (Norwalk, CT, USA) at a wavelength of 750 nm after 30 minutes at room temperature and protected from light. The total phenolic content was expressed in milligrams of gallic acid equivalent per mL of sample (mgGAE/mL).

#### 2.3.2. Total Flavonoid Content

The method described by Chang et al. [19] was used with some modifications to determine the total flavonoid content. To a 25 mL flask were added 500 *μ*L of the sample and 500 *μ*L of AlCl_3_ 0.5%, and the volume was completed with water. The reading was done in a Lambda 35 Perkin Elmer UV-VIS spectrophotometer (Norwalk, CT, USA) at a wavelength of 415 nm after being incubated for 40 minutes at room temperature and protected from light. The results were expressed in rutin mg equivalents/g (mgRE/g).

### 2.4. HPLC-DAD Assay

Chromatographic analyses were performed using a Prominence Shimadzu® HPLC system (Kyoto, Japan) equipped with an LC-20AT pump, SIL-20A autoinjector, SPD-20AT detector, CTO-20A column oven, and LC Solution V. 1 : 24 SP1 software. The chromatographic separation was performed in a RP-C18 column Hypersil C18 Thermo-Scientific (250 × 4.0 mm, 5 *μ*m) using a mobile phase of 5% (v/v) acetonitrile (solvent A) or 50% (v/v) acetonitrile (solvent B) containing 0.05% (v/v) phosphoric acid (85%), with a 1 mL/min flow rate, Diode Array Detector (DAD) at 280 nm and 340 nm, and an injection volume of 40 *μ*L. The gradient was programmed as follows: solvent A was maintained at 90%, and solvent B was maintained at 10% within the first 14 minutes. B increased linearly from 10% to 15% over 14–20 minutes, and this condition was maintained for four minutes. B increased linearly from 15% to 70% over 24–40 minutes and this condition was maintained for four minutes. The chromatographic parameters were adapted from Yang et al. [20] method.

To identify and suggest the presence of components in the leaf extract of* C. xanthocarpa*, peak retention times were studied and a thorough comparison between sample and standard references was made. The standard compounds used were gallic acid (200 *μ*g/mL), chlorogenic acid (200 *μ*g/mL), quercetin (200 *μ*g/mL), luteolin (100 *μ*g/mL), isoquercetin (100 *μ*g/mL), quercitrin (100 *μ*g/mL), theobromine (35 *μ*g/mL), caffeic acid (35 *μ*g/mL), and caffeine (35 *μ*g/mL), prepared with ethanol 50% (v/v). Extract samples and references standards were analyzed in triplicate.

### 2.5. Evaluation of Blood Pressure and Possible Action Mechanisms of the* Campomanesia xanthocarpa* Extract

#### 2.5.1. Animals

Male Wistar rats were used that were three months old and purchased from Universidade Federal de Santa Maria. They were kept in cages with a controlled temperature (22°C), 12 hour dark/light cycle, food, and water ad libitum.

The experimental protocols followed the International Principles for Research Involving Animals (Geneva), Brazilian legislation by Law No. 11.794/2008 (Procedures for the Scientific Use of Animals), and Decree 24.645/34 (animal rights) and were approved by the Ethics Committee on Animal Use (ECAU) of the university (Protocol 017/2014).

After all of the protocols, the animals were sacrificed using the guillotine while still under the effects of the anesthesia.

#### 2.5.2. Hemodynamic Parameters

The rats were submitted to surgery for catheterization of the carotid artery (to measure the hemodynamic parameters) and the jugular vein (for extract and drugs administration), with polyethylene catheters (PE10 Clay-Adams) filled with heparinized saline (50 IU/mL), under anesthesia with urethane (1.4 g/kg, i.p.), which was assessed by responsiveness to painful stimuli and supplemented when necessary [21]. The arterial catheter was connected to a pressure transducer coupled to an analog digital converter (Biopac Systems MP150, Inc.; CA).

#### 2.5.3. Extract Curve

For evaluation of the extract effects on hemodynamic parameters, a rising curve was done, after 30 minutes of stabilization. A total of 25, 50, 75, 100, 125, 150, 175, and 200 mg/kg of extract cumulative were administered, with a volume of 0.2 mL of saline; each dose was administered 15 minutes after the other (*n* = 8). Systolic blood pressure (SBP), diastolic blood pressure (DBP), and heart rate (HR) were recorded over approximately 3 hours.

#### 2.5.4. Evaluation of Possible Action Mechanisms of the Extract

For the investigation of mechanisms involved in the extract hypotensive action, certain drugs were tested after 30 minutes of stabilization: N_*ω*_-nitro-L-arginine methyl ester (L-NAME), an nitric oxide (NO) synthesis inhibitor (30 mg/kg, *n* = 6); losartan, AT1 receptor antagonist of angiotensin II (10 mg/kg, *n* = 6); and hexamethonium, a ganglionic blocker (20 mg/kg, *n* = 6) [21–23]. After drug administration, a new stabilization period was conducted, and then the extract (5 to 100 mg/kg) was administered, with the exception of the L-NAME protocol that used doses of 5 to 25 mg/kg because 50 mg/kg of the extract after administration of this drug caused a sharp decrease in blood pressure, inducing some animals' death. Hemodynamic records of SBP, DBP, and HR were taken.

#### 2.5.5. Drugs

All drugs and reagents used were purchased from Sigma Chemical Co. (St. Louis, MO, United States).

### 2.6. Molecular Modeling Studies

Computational studies were performed with chlorogenic acid and quercetin, components determined in aqueous extract, to obtain information which aided in the understanding of the biological activity. Density functional theory (DFT) B3LYP/6-311G^*∗*^, based in gas phase methodology, evaluable in Spartan'08 for Windows software (Wavefunction Inc., Irvine, United States) was used for geometry optimization and conformational analysis. The geometry of compounds was optimized followed by submission to systematic conformational analysis with a torsion angle increment set of 30° in the range of 0–360°. The lowest energy conformer for chemical structure was saved in a mol^2^ file before use in the docking studies.

The structure of angiotensin II type 1 receptor, AT1R encoded PDB ID: 4YAY [24], was downloaded from the protein data bank (PDB) before performing the docking studies, and this 3D structure was prepared by removing the water molecules and adding polar hydrogens using Autodock Tools 1.5.6 [25]. Using iGemdock software [26], docking studies were performed in which the individual binding site of chlorogenic acid and quercetin were assessed and submitted to docking in the active site of the AT1R. Docking calculations were performed at drug screening docking accuracy setting with GA parameters set for population size, generation, and number of solutions as 200, 70, and 3, respectively, with a Gemdock score function of hydrophobic and electrostatic (1 : 1 preference). iGemdock software was used to infer the pharmacological interactions between the biological receptors and compounds studied.

### 2.7. Statistical Analysis

Data were expressed as the mean ± standard deviation (SD), values were analyzed by one-way ANOVA for repeated measures and Tukey's post hoc, and *p* < 0.05 was considered significant.

## 3. Results

### 3.1. Characterization of Plant and Extracts

The value of the total phenolic compounds found in the analysis of* C. xanthocarpa* extract was 3.7360 mgGAE/mL (relative standard deviation 2.87%), and the flavonoid content was 2.5070 mgRE/g (relative standard deviation 3.45%).

### 3.2. HPLC-DAD Analysis

**Figure 1:**
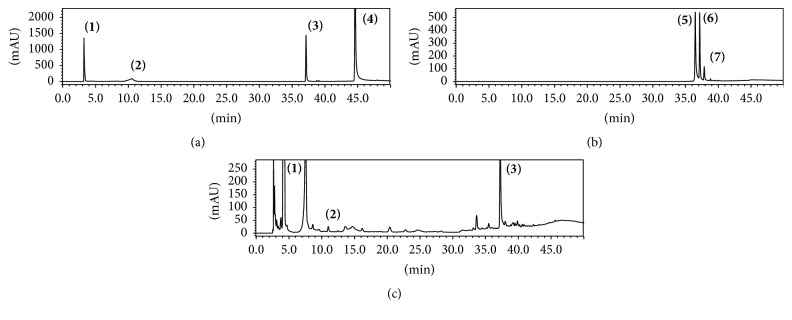
Representative chromatogram obtained in the analysis by HPLC-DAD wherein (a) phenolic standards are (1) gallic acid, (2) chlorogenic acid, (3) quercetin, and (4) luteolin; (b) phenolic standards are (5) vitexin, (6) isoquercetin, and (7) quercitrin; and (c)* C. xanthocarpa* aqueous extract, detection at 340 nm.

**Figure 2:**
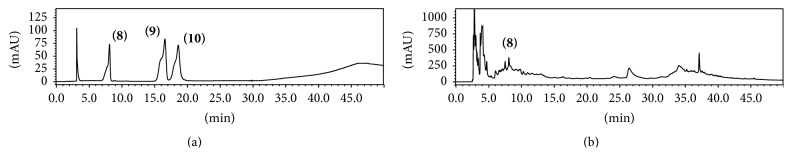
Representative chromatogram obtained in the analysis by HPLC-DAD wherein (a) xanthine standards and caffeic acid are (8) theobromine, (9) caffeic acid, and (10) caffeine and (b)* C. xanthocarpa* aqueous extract, detection at 280 nm.

**Figure 3:**
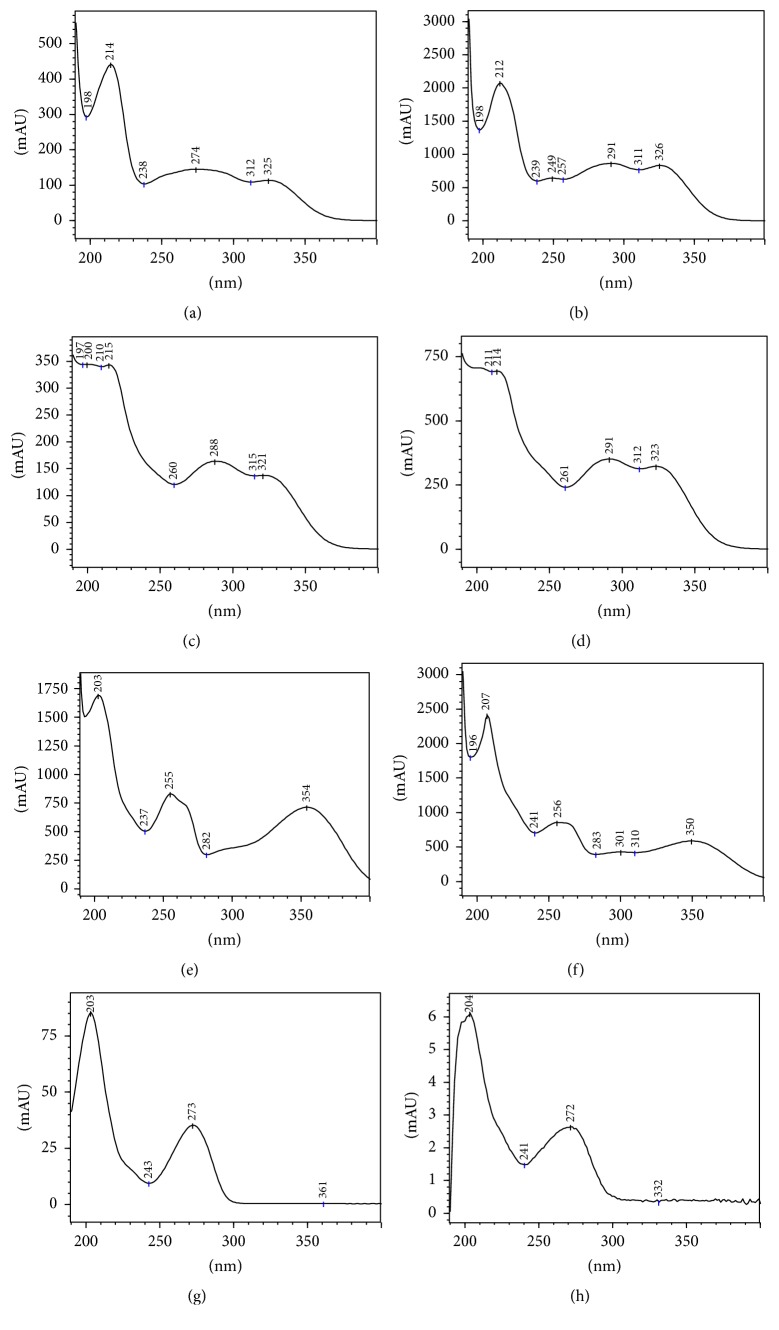
UV spectra obtained from DAD analysis applied to each chromatographic peak for standard references and* C. xanthocarpa* extract. (a) UV spectra for compound 1 (gallic acid) in standard reference; (b) UV spectra for compound 1 (gallic acid) in the extract; (c) UV spectra for compound 2 (chlorogenic acid) in standard reference; (d) UV spectra for compound 2 (chlorogenic acid) in the extract; (e) UV spectra for compound 3 (quercetin) in standard reference; (f) UV spectra for compound 3 (quercetin) in the extract; (g) UV spectra for compound 8 (theobromine) in standard reference; and (h) UV spectra for compound 8 (theobromine) in the extract.

The results showed the presence of many compounds in the crude extract of* C. xanthocarpa*. In Figure 1, it is possible to observe the complexity of plant matrix, whose constituents are being detected along the chromatographic run; in this case, the detection at 340 nm was focused in phenolic compounds. Compared to the reference standards, the similarity between retention times, besides UV spectra for chromatographic peaks, is indicative for the presence of gallic acid (compound 1), chlorogenic acid (compound 2), and quercetin (compound 3). In another assay (Figure 2), aiming to evaluate the xanthine derivatives and caffeic acid by performing the detection at 280 nm, we suggest the presence of theobromine (compound 8) in the medicinal plant studied. It is important to mention that the peaks were evaluated using UV-DAD detection, whose UV profile data were thoroughly studied in order to confirm the chemical composition suggested (Figure 3).

### 3.3. Evaluation of Blood Pressure and Possible Action Mechanisms of* C. xanthocarpa* Extract

#### 3.3.1. Evaluation of Hemodynamic Parameters in the Dosage Curve of* C. xanthocarpa* Extract

**Figure 4:**
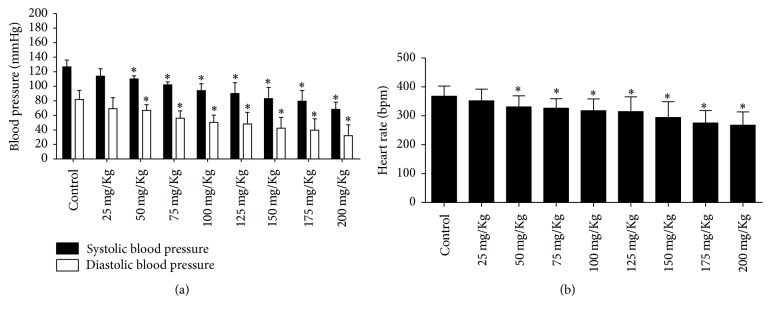
Evaluation of hemodynamic parameters in the dosage curve of* C. xanthocarpa* extract. (a) Register of the systolic and diastolic blood pressure with different administration of extract doses. (b) Heart rate during curve dosage of* C. xanthocarpa* extract. ^*∗*^*p* < 0.05 versus control (*n* = 8).

In the dose-administration curve, it can be observed that blood pressure decreased with the extract from 50 mg/kg in a dose-dependent manner. SBP and DBP decreased from 126.71 ± 9.26 to 68.14 ± 9.99 mmHg and from 81.85 ± 12.58 to 32.14 ± 14.89 mmHg, respectively (Figure 4(a)). Heart rate also decreased in the presence of the extract from 50 mg/kg to 200 mg/kg (Figure 4(b)).

#### 3.3.2. Evaluation of the Possible Action Mechanism of* C. xanthocarpa* Extract

**Figure 5:**
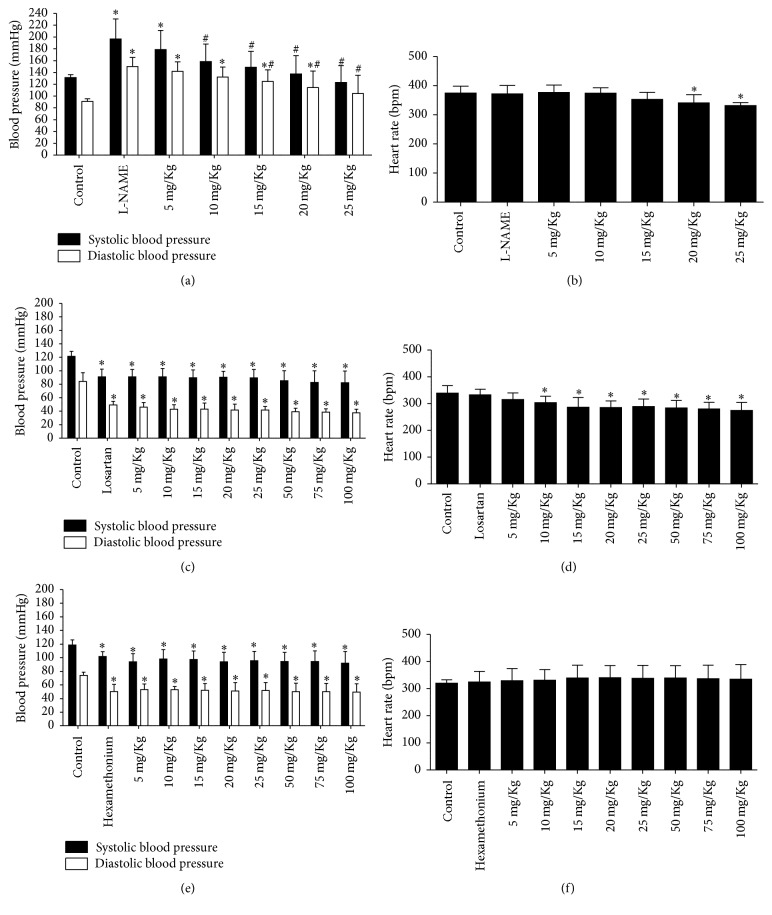
Evaluation of hemodynamic parameters in rats treated with* C. xanthocarpa* extract in the presence of different drug administration. (a) Register of the systolic and diastolic blood pressure with extract in the presence of L-NAME (30 mg/kg). (b) Register of heart rate during L-NAME protocol. (c) Register of the systolic and diastolic blood pressure with extract in the presence of losartan (20 mg/kg). (d) Register of heart rate during losartan protocol. (e) Register of the systolic and diastolic blood pressure with extract in the presence of hexamethonium (10 mg/kg). (f) Register of heart rate during hexamethonium protocol. ^*∗*^*p* < 0.05 versus control and ^#^*p* < 0.05 versus drug (*n* = 6).

As expected, L-NAME (Figure 5(a)) caused a hypertensive peak of 73.2–59 mmHg (SBP–DBP), but when the extract was administered the blood pressure decreased to normal levels, suggesting that NO synthase mechanism is not involved in the hypotensive effect of the extract. The heart rate decreased in the presence of L-NAME after administration of the extract at doses of 20 and 25 mg/kg (Figure 5(b)).

Losartan (Figure 5(c)) and hexamethonium (Figure 5(e)) decreased blood pressure from 121.4 ± 7.56 × 84.2 ± 12.87 to 91.2 ± 11.23 × 49.4 ± 5.27 mmHg and 118.75 ± 7.41 × 74 ± 4.76 to 101.75 ± 7.18 × 50.25 ± 10.53 mmHg, respectively. After administration of these drugs, extract administration occurred, and no alterations to blood pressure were detected; this may mean that the hypotensive action of the extract can be implicated in the AT1R mechanism and sympathetic ganglionic blockade. In the losartan protocol, with 10 mg/kg of the extract, the heart rate decreased from 339 ± 28.41 to 274.4 ± 30.16 (Figure 5(d)). With hexamethonium, the heart rate was not altered (Figure 5(f)).

### 3.4. Molecular Modeling Studies

**Figure 6:**
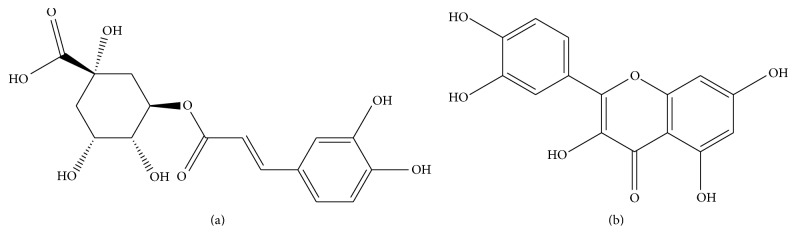
Chemical structure of chlorogenic acid (a) and quercetin (b).

**Figure 7:**
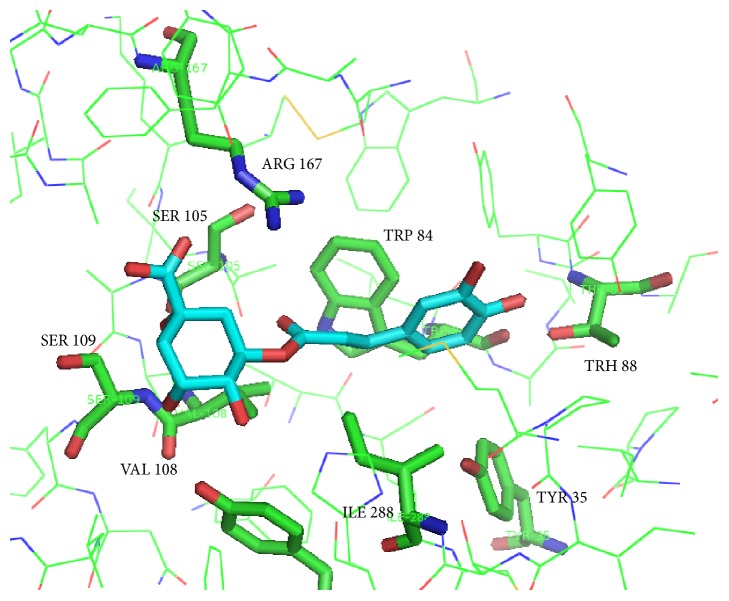
Binding of chlorogenic acid and quercetin in the active site of AT1R (PDB: 4YAY). Graphic visualization obtained using PyMOL (v.0.99).

Molecular modeling studies were performed on two of the main components of aqueous extract from* C. xanthocarpa*: chlorogenic acid and quercetin (Figure 6). Docking was run using the conformer of minimal energy of chemical structure generated by Spartan, and results from the interaction are shown in Table 1. The active interactions between the compound and AT1R are shown in Figure 7.

## 4. Discussion

Many phytochemical studies provide evidence of the presence of phenolic compounds and flavonoids in the* Campomanesia* genus [27, 28]. Some demonstrate higher values than the findings in our study, which can be justified by several factors, including seasonality, the type of soil in which the plant was grown, water availability, nutrient supply, minerals, development stage, temperature, ultraviolet radiation, induction by mechanical action stimuli, or pathogens [29].

Because phenolic compounds and flavonoids are known to have many pharmacological activities, the* C. xanthocarpa* extract may have promising biological effects. The phenolic compounds gallic acid, quercetin, and chlorogenic acid have several biologically related uses, such as analgesic, antidiabetic, anti-inflammatory, antiobesity, antioxidant, cardioprotective, hypotensive, and neuroprotective effects [30–33]. These substances, observed in our study, have already been shown in other studies with plants of the Myrtaceae family [34–36].

Phenolic compounds, mainly flavonoids, beyond other biological activities, act as antioxidants, not only for their ability to donate hydrogen or electrons but also because of their stable intermediate radical, which prevents the oxidation of several food molecules, particularly lipids [28]. The well-recognized antioxidant properties of flavonoids resulted in the interest in their potential role in prevention of cardiovascular diseases [37].

The antioxidant properties of natural products depend not only on polyphenol content but also on type. For instance, quercetin and catechin demonstrate the greatest antioxidant properties in vitro [38]. Studies have shown the effect of quercetin on the prevention and treatment of cardiovascular diseases, cancer, and renal and hepatic insufficiency [39]. These actions could be linked to the ability of the maintenance of vascular integrity due to the sequestration of free radicals and the inhibition of lipid peroxidation, lipoxygenase, cyclooxygenase, and phospholipase A2 activity. They also inhibit LDL oxidation, platelet aggregation, and C kinase protein activity and promote vasodilatation [31]. Thus, the high content of quercetin in* C. xanthocarpa* extract can be related to its hypotensive action.

Chlorogenic acid and gallic acid are present in the studied extract. These substances are phenolic compounds with important biological properties beyond antioxidant action; they also have insulin-sensitizing activities, reduce hyperglycemia via several mechanisms, and have a cardioprotective effect [32, 40].

Other secondary plant metabolites are alkaloids, the most diverse group found in living organisms with an array of structure types, biosynthetic pathways, and pharmacological activities [41]. The alkaloid theobromine (3,7-dimethylxanthine; 3,7-DMX) derived from caffeine metabolism has a moderate diuretic effect on the kidneys, causes mild cardiac stimulation, and acts on the central nervous system and cardiovascular, renal, and digestive systems [42, 43]. It also increases serum HDL-cholesterol concentrations that may be important in CVD prevention [44]. However, this is the first time that this compound is reported in* C. xanthocarpa*; its main existence is related to other species such as* Paullinia cupana, Ilex paraguariensis, Coffea arabica, Theobroma cacao*, and* Camelia sinensis* [45–48].

The dose nature of effects of the extract on blood pressure and heart rate of the rats suggests a cumulative action of the active substances present in the leaves of the plant. Thus, we can attribute the hypotensive effect of* C. xanthocarpa* to its phenolic and alkaloid compounds. Despite some reports, the present study is the first that describes the hypotensive activity of this extract, which is extremely important because the incidence of hypertension and use of medicinal plants have been increasing worldwide, generating future potential natural therapies.

To investigate the mechanism of the hypotensive action of* C. xanthocarpa*, drugs were administered before the extract. L-NAME is an analog of arginine competing for NO synthase and causes a hypertensive peak, resulting in the gross inhibition of NO synthesis [49]. It is widely used in experiments with rats as a model of systemic hypertension [50, 51]. Thus, that the extract decreased blood pressure after administration of this drug suggests that NO synthase mechanism is not involved in the hypotensive effect.

Losartan acts through blocking the renin-angiotensin system by selectively antagonizing the receptors for angiotensin II, subtype AT1R. Previous evidence shows that the blockade of angiotensin AT1R results in reduced oxidative stress, inflammatory markers, and fibrinolysis inhibition [52].

Hexamethonium is potent and essentially devoid of neuromuscular blocking activity by a nicotinic receptor-blocking agent that blocks ion channels of the autonomic ganglia, resulting in blockage of the outputs of the sympathetic pathways [53, 54]. Due to its charged structure, hexamethonium peripherally administered is presumed not to enter the brain and therefore is commonly used to distinguish peripheral ganglionic responses in vivo from those that arise centrally [53].

As the extract did not alter blood pressure after administration of losartan and hexamethonium, it can be suggested that the hypotensive action of the extract implicates the AT1R mechanism and sympathetic ganglionic blockade.

The docking procedure was aimed to identify individual poses of chlorogenic acid and quercetin, components of* C. xanthocarpa* extract, that may bind to the AT1R active site. Chlorogenic acid was chosen for the docking analysis for its recognized antihypertensive activity [33], and quercetin was selected because this compound showed downregulation of the AT1R in the kidneys [55].

The empirical scoring function of iGemDOCK is the estimated sum total of Van der Waals, H-bond, and electrostatic energy. In this case, the comparison of chlorogenic acid ligand poses chlorogenic acid and quercetin structures revealed similar conformations and conserved molecular recognition modes for antagonists and inverse agonists toward AT1R [24]. Chlorogenic acid and quercetin showed an affinity energy of −91.44 and 88.15 Kcal mol^−1^. Chlorogenic acid showed Van der Waals, H-bond, and electrostatic interaction values of −76.11, −13.35, and −1.98 Kcal mol^−1^, respectively, and quercetin showed only Van der Walls and H-bond at −62.01 and 26.14 Kcal mol^−1^, respectively. No electrostatic interaction of quercetin and AT1R was observed.

According to the data of docking experiments, main interactions occur with the residues TYR 35, TRP 84, THR 88, SER 105, VAL 108, SER 109, ARG 167, and ILE 288 (Figure 7). After the postscreening analysis using residue consensus analysis, ARG 167 was detected as the main residue involved in this ligand-receptor binding (with a* Z*-score of −1.12 and WPharma of 1.00) to chlorogenic acid only. The ARG 167 and TRP 84 residues are the same observed in the docking study Zhang et al. [24] performed, suggesting the active site of AT1R studied.

A model generated from chlorogenic acid suggests the interaction of H-bond, Van der Waals, and electrostatic types with ARG 167. The carboxylic acid attached to the cyclohexane ring of chlorogenic acid's chemical structure was located in the same spatial region as determined by the docking of the same moiety from the telmisartan or tetrazole ring from losartan [24]. The main type of interaction was a Van der Waals interaction with the TYR 35, TRP 84, SER 105, VAL 108, ARG 167, and ILE 288 residues. In the case of chlorogenic acid, there is a molecular distance of 2.82 Å between ARG 167 and a carboxylic acid moiety attached to the hexane ring and the 2.82 Å of hydrogen from hydroxyl group with THR 88, and 3.01 Å of hydroxyl and SER 109 of AT1R were observed.

Some main interactions of the quercetin ligand and AT1R were H-bonds with ARG 167 and Van der Waals with the THR 84 and ILE 288 residues. Quercetin showed an interaction with the hydroxyl moiety attached to aromatic ring TYR 35; this group has distances of 2.8 and 3.10 Å between hydroxyl groups and the flavonol nucleus. Carbonyl moiety from flavonols showed a molecular distance of 2.70 Å of the nitrogen atom from the tetrazole ring of ARG 167. These data suggest that chlorogenic acid and quercetin, which are important components in the plant extract, may be the compounds causing the interaction of cardiac AT1R.

Based on these results, it can be concluded that the aqueous extract of* C. xanthocarpa* presents expressive phenolic and flavonoid content. Additionally, the presence of chlorogenic and gallic acid, quercetin, and theobromine in the extract was determined. It was hypothesized that two constituents of the plant may be attributed to its AT1R interaction. These substances already have a clinical potential described that can assist in the investigation of the pharmacological properties of this plant. However, the acute administration of the aqueous extract of* C. xanthocarpa* has a dose-dependent hypotensive effect in normotensive rats, and we suggest that this action may be mediated through the renin-angiotensin system by AT1R blockade and a sympathetic autonomic response.

## Figures and Tables

**Table 1:** Central pharmacological interactions (Van der Waals, H-bond, and electrostatic) of compounds and residues involved in the binding site of ACE-I with normal and applying the residues consensus analysis.

Compounds	TYR 35	TRP 84	THR 88	SER 105	VAL 108	SER 109	ARG^*∗*^ 167	ILE 288
*Chlorogenic acid*								
Van der Waals	−5.5	−13.0	0.0	−5.7	−7.2	0.0	−6.3	−6.9
H-Bond	0.0	0.0	−2.5	0.0	0.0	−4.2	−4.1	0.0
Electrostatic	0.0	0.0	0.0	0.0	0.0	0.0	−1.9	0.0

*Quercetin*								
Van der Waals	−4.2	−19.0	0.0	−6.9	−0.2	−5.1	0.0	−8.2
H-Bond	−5.0	0.0	0.0	−2.1	0.0	0.0	−15.7	0.0
Electrostatic	0.0	0.0	0.0	0.0	0.0	0.0	0.0	0.0

^*∗*^Confirmed by residue consensus analysis.
